# Destruction of Vowel Space Area in Patients with Dysphagia after Stroke

**DOI:** 10.3390/ijerph192013301

**Published:** 2022-10-15

**Authors:** Min Kyu Choi, Seung Don Yoo, Eo Jin Park

**Affiliations:** 1 Department of Rehabilitation Medicine, Kyung Hee University Hospital at Gangdong, Seoul 05278, Korea; 2 Department of Medicine, AgeTech-Service Convergence Major, Kyung Hee University, Seoul 02447, Korea

**Keywords:** destruction of vowel space, dysphagia, dysarthria, stroke

## Abstract

Dysphagia is associated with dysarthria in stroke patients. Vowel space decreases in stroke patients with dysarthria; destruction of the vowel space is often observed. We determined the correlation of destruction of acoustic vowel space with dysphagia in stroke patients. Seventy-four individuals with dysphagia and dysarthria who had experienced stroke were enrolled. For /a/, /ae/, /i/, and /u/ vowels, we determined formant parameter (it reflects vocal tract resonance frequency as a two-dimensional coordinate point), formant centralization ratio (FCR), and quadrilateral vowel space area (VSA). Swallowing function was assessed using the videofluoroscopic dysphagia scale (VDS) during videofluoroscopic swallowing studies. Pearson’s correlation and linear regression were used to determine the correlation between VSA, FCR, and VDS. Subgroups were created based on VSA; vowel space destruction groups were compared using ANOVA and Scheffe’s test. VSA and FCR were negatively and positively correlated with VDS, respectively. Groups were separated based on mean and standard deviation of VSA. One-way ANOVA revealed significant differences in VDS, FCR, and age between the VSA groups and no significant differences in VDS between mild and moderate VSA reduction and vowel space destruction groups. VSA and FCR values correlated with swallowing function. Vowel space destruction has characteristics similar to VSA reduction at a moderate-to-severe degree and has utility as an indicator of dysphagia severity.

## 1. Introduction

Dysphagia can cause malnutrition, dehydration, and aspiration pneumonitis; therefore, appropriate screening and treatment are required for patients with stroke. Dysphagia and dysarthria are common in approximately 40% of patients with stroke, and co-occurrence is reported in approximately 28% of them [1]. This may be due to the fact that swallowing and speech share a number of pharyngeal, oral, and laryngeal structures [2]. Post-stroke dysphagia is defined as pharyngeal and oral difficulties caused by alterations in the strength, mobility, and sensitivity of laryngeal and orofacial muscles [3]. Dysarthria is related to promotion of mastication and preparation of bolus in the oral phase, as well as the protection of airways in the pharyngeal phase [4]. Dysarthria has also been reported to be an indicator of oral phase problems in swallowing disorders [5,6].

**Figure 1:**
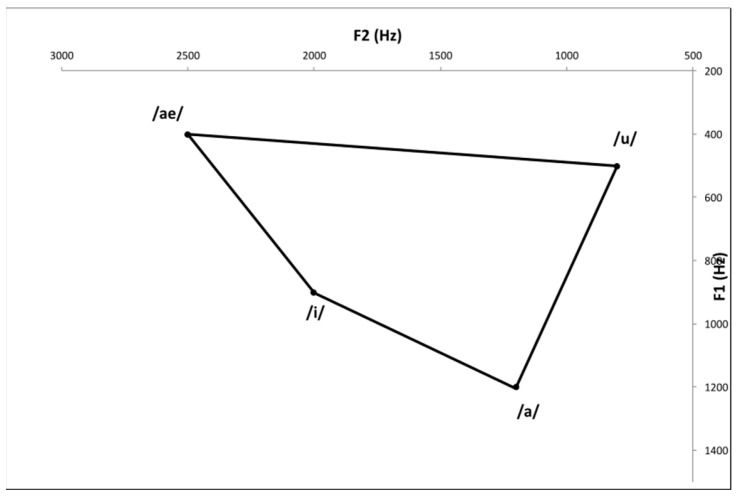
Normal acoustic vowel space.

**Figure 2:**
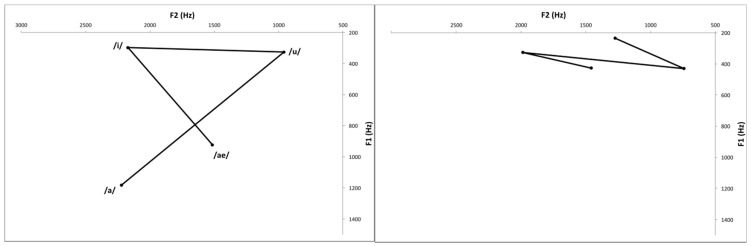
Distortion of acoustic vowel space.

Reduced excursion and velocity of lip, lingual, and jaw motions, as well as aberrant motion timing, are implicated in speech problems associated with vowel production deficiencies [7]. The extrinsic muscles of the tongue, such as the styloglossus, palatoglossus, genioglossus, and hyoglossus are important for vowel production. The quadrilateral vowel space created by the motion of these muscles can be measured as an objective acoustical evaluation [8,9]. To evaluate vowel production, the quadrilateral vowel space can be measured as an objective acoustical evaluation. The vowel space area (VSA) is related to intelligibility and proper articulation of vowels [10,11,12]. Centralization of formant frequency has also been shown in vowel production of patients with dysarthria [13]. The main feature of dysarthria is a narrowed range of articulation, which makes it difficult to achieve the desired position and intensity of vocal fold contractions. Vowel formant concentration could result from this undershoot, and formant centralization ratio (FCR) indicates the degree of this centralization [14]. Additionally, in some cases, it is impossible to measure the VSA because the area of the vowel space of the stroke patient is severely distorted (Figure 1 and Figure 2). Therefore, it is necessary to evaluate the meaning of vowel space destruction for dysphagia, which can help evaluate swallowing function through objective acoustic assessment and provide evidence on the need for rehabilitation.

In this study, a correlation analysis of VSA, FCR values, and videofluoroscopic dysphagia scale (VDS) scores was performed to determine whether the acoustic vowel space was associated with swallowing function in stroke patients. We also investigated the relationship between vowel space destruction and the severity of dysphagia in patients with stroke.

## 2. Materials and Methods

### 2.1. Participants

We retrospectively gathered data on stroke patients with dysphagia and dysarthria who were admitted to the Kyung Hee University Hospital at Gangdong between March 2018 and March 2020. The patients with subacute stroke more than 2 weeks and within 1 month after stroke onset were chosen for vowel space assessment and videofluoroscopic swallowing study (VFSS). Dysarthria was diagnosed using the Urimal Test of Articulation and Phonation (U-TAP) [15]. The inclusion criteria were as follows: only one stroke episode, must cooperate with the voice examination, must score ≥20 on the Mini-Mental State Examination (MMSE), and must be a native Korean speaker. Hearing loss or aphasia, speech or swallowing difficulties caused by illnesses other than stroke, and anatomical abnormalities in the articulation organs were all considered exclusion criteria. The institutional review board (IRB) of Kyung Hee University Hospital at Gangdong approved the study protocol (IRB approval number: 2022-03-013).

### 2.2. Videofluoroscopic Dysphagia Scale

Videofluoroscopic swallowing study was conducted by two rehabilitation physicians and a radiologist. Each patient consumed yogurt, liquid, thin rice porridge, rice, or porridge with barium contrast medium. The tests were recorded and examined by two rehabilitation physicians who then calculated the VDS scores. VDS evaluates dysphagia by scoring each item in the oral phase and pharyngeal phases through the VFSS results. VDS is a sensitive and specific method for quantifying the severity of dysphagia that persists after stroke, based on the high reliability of VFSS [16].

The VDS consists of 14 items, with the oral phases represented by premature bolus loss, bolus formation, lip closure, apraxia, mastication, and tongue-palate contact. Pharyngeal phases are represented by vallecular residue, pharyngeal swallow triggering, laryngeal elevation, coating of the pharyngeal wall, pyriform sinus residue, and pharyngeal transit time [17]. The total VDS score is a numeric value between 0–100, with a higher score indicating a worse swallowing function.

### 2.3. VSA and FCR

Praat is a computer application often used in speech acoustic analysis [18]. All speech samples were tested using Praat (version 4.4.22, Institute of Phonetic Sciences, Amsterdam, The Netherlands) at a sampling rate of 22,050 Hz, and a formant analysis was employed. A Shure SM48 microphone was used in conjunction with a Visi-Pitch IV to capture the audio. Praat quantifies acoustic properties, such as jitter and shimmer, and represents speech functions as discrete sound component spectra [19]. The formant parameter reflects vocal tract resonance frequency as a two-dimensional coordinate point. The patients rehearsed each vowel for more than 3 s, three times before testing. The number of formants is related to the size and morphology of the oral cavity. The first formant (F1) is connected to the upper and lower locations of the tongue, as well as the constriction of the vocal tract and capacity of the pharyngeal cavity. The second formant (F2) was connected to the positions of the tongue’s anterior and posterior surfaces, as well as the length of the oral cavity.

Vowel surface area represents the size of the square formed by the four corner vowels (/a/, /i/, /u/, and /ae/) when projected at the first two formant frequencies. The FCR is calculated using a technique that standardizes individual variable variations to account for instabilities in the VSA result, which is used to measure articulatory movements and related statistical insensitivity [14]. When vowel centralization increases, the FCR increases; when vowel expansion increases, the FCR decreases. The FCR and VSA were calculated according to the following formulas [14,20]:

VSA = ½|[(F2/i/×F1/ae/ + F2/ae/×F1/a/ + F2/a/×F1/u/ + F2/u/×F1/i/) − (F1/i/×F2/ae/ + F1/ae/×F2/a/ + F1/a/×F2/u/ + F1/u/×F2/i/)]|

FCR = (F2/u/ + F2/a/ + F1/i/ + F1/u/)/(F2/i/ + F1/a/)

### 2.4. Statistical Analysis

Statistical analyses were performed using SPSS version 25.0. Pearson’s correlation coefficients were used to determine the relationship between VSA, FCR, and VDS. The VDS scores were assessed using linear regression analysis of the VSA and FCR.

Using the mean and standard deviation of the VSA, patients in group A had VSA values below -1 standard deviation and were defined as a group with severe VSA.

Patients in Group B had VSA values above -1 standard deviation and below the mean value and were defined as a group with moderate to severe reduced VSA. Patients in group C had VSA values above the mean and below +1 standard deviation and were defined as a group with reduced VSA with a mild to moderate degree. Patients in group D had VSA values above +1 standard deviation and were defined as having mild VSA. Group E is a group with destruction of the vowel space. One-way analysis of variance (ANOVA) of VDS, FCR, and age among the five groups was performed. Any significant differences post-test were analyzed using the Scheffe’s test. Significance level for all the statistical tests was set at *p* < 0.05.

## 3. Results

### 3.1. Participants

This study included 74 stroke patients with dysarthria and dysphagia. The study population included 45 male and 29 female participants with a mean age of 67.38 ± 12.63 years. Forty-seven cases of ischemic stroke and 27 cases of hemorrhagic stroke were included in the study population. The mean MMSE score, VSA, modified Barthel index, VDS, and FCR values are shown in Table 1.

### 3.2. Correlation of VSA and FCR with VDS

Vowel surface area was significantly associated with VDS (r = −0.760, *p* = 0.001) negatively, whereas FCR was significantly associated with VDS (r = 0.417, *p* = 0.001) positively, and age had a significant positive correlation with VDS (r = 0.238, *p* = 0.041) (Table 2). Multivariate linear regression analysis showed that VSA was a predictor of VDS (R^2^ = 0.571) (standardized coefficient = −0.760, *p* = 0.001) (Table 3).

### 3.3. Comparison of VDS, FCR, and Age among Subgroups Dvided by VSA

**Figure 3:**
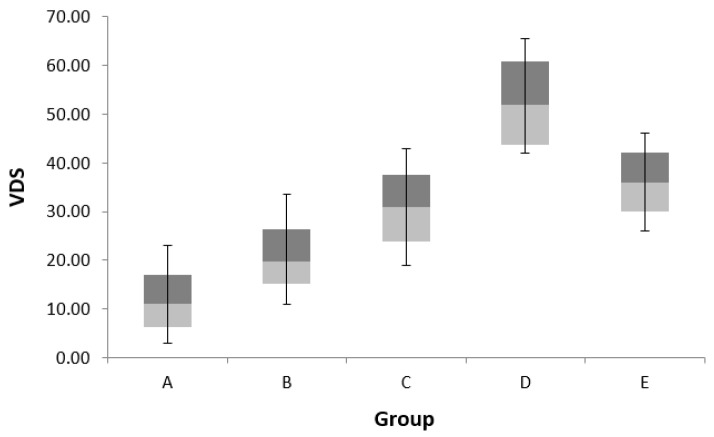
Comparison of values of videofluoroscopic dysphagia scale in each group.

One-way ANOVA between the groups divided by VSA showed significant differences in VDS, FCR, and age. In a post hoc study, VDS was higher in group D than in group A. There were no significant differences between groups C and E. The FCR showed higher values in group E than in groups A, B, C, and D, but age did not show a significant difference in the post hoc analysis (Table 4 and Figure 3).

## 4. Discussion

In this study, we found that the VSA and FCR obtained through vowel quadrilateral evaluation were related to swallowing function. The smaller the VSA, the higher the severity of dysphagia; the higher the FCR, the higher the severity of dysphagia. Vowel surface area is a significant factor in dysphagia severity. These results are consistent with our previous findings [6]. Additionally, in the group divided according to VSA, the VDS showed a significant difference, and the group with a lower VSA tended to have higher dysphagia severity. The group with vowel space destruction showed no significant difference in dysphagia severity compared with the group with mild-to-moderately reduced VSA. However, all the dysphagia severities were significantly different from those of the other groups. Given these results, the destruction of vowel space can be considered to correspond to a moderate to severe decrease in VSA.

Dysarthria is a neuromotor condition caused by anomalies in the speed, strength, stability, tone, range, or accuracy of movements necessary for speech control [21]. Dysarthria affects articulation, phonation, breathing, nasality, and the prosody of speech, thereby impairing audibility, intelligibility, naturalness, and efficiency. Dysarthria is related to oral phase problems, which are initial indicators of dysphagia [22]. These oral-phase difficulties include diminished sensation in the lip, face, cheek, palate, tongue, tongue weakness, decreased tension in the mouth floor, and anterior hyoid protrusion. These issues may result in buccal, lingual, and palatal stasis, floor-of-mouth stasis, reduced hyoid elevation, difficulties in posterior bolus transport, manipulation of boluses, cohesiveness, and vallecula and pyriform stasis. Additionally, they may alter the pharyngeal phase, resulting in inadequate vocal fold laryngeal vestibule closure, insufficient clearing of laryngeal vestibule residues, and aspiration [23,24].

FCR and VSA are measures of vowel pronunciation accuracy that show an individual’s ability to control tongue movement. A reduced VSA and higher FCR are associated with tongue and jaw movement limitations. It has been shown that tongue movement problems are substantially related to dysarthria and dysphagia [25]. In our previous study, acoustic vowel space evaluation was found to be a useful tool for evaluating dysarthria, and it showed a correlation between dysarthria and dysphagia [6]. However, there are many patients with severe distortion of vowels whose VSA cannot be measured because the shape of the vowel square is broken. It may be clinically important to predict the severity of dysphagia in patients with deformed shapes during vowel quadrilateral examination. Assessing dysphagia risk is crucial for avoiding complications (e.g., aspiration pneumonia) and developing rehabilitation objectives and programs for patients.

Thus, when patients complain of dysarthria following a stroke, a speech production evaluation that includes vowel space measurements can be used to predict dysphagia severity. Speech therapy may also be beneficial in improving swallowing difficulties and preventing complications, such as aspiration pneumonia. Additionally, since the National Institutes of Health Stroke Scale is used as the first standard screening tool for stroke patients, dysarthria evaluation provides a simple way to evaluate whether additional dysphagia evaluation is warranted.

This study had limitations, owing to the number of participants being small and the nature of the study being retrospective. In the group with broken shape, detailed classification according to the distorted shape (e.g., high and low vowel inversions) and anterior and posterior vowel inversions were not conducted; therefore, it is unclear whether dysarthria is related to dysphagia severity according to shape. In addition, patients with ischemic and hemorrhagic strokes were mixed together, and the location of stroke lesions varied, so the test group was not homogenous. Therefore, further large-scale prospective studies are warranted by dividing the patient group into ischemic stroke and hemorrhagic stroke.

## 5. Conclusions

VSA and FCR obtained through the vowel quadrilateral test were useful parameters related to the severity of dysphagia, and patients with a broken vowel quadrilateral shape had mild to moderate severity of dysphagia.

## Figures and Tables

**Table 1:** Demographic and clinical data of patients.

Characteristic	Value
Age (yr)	67.38 ± 12.63
Sex	
Male	45 (60.80)
Female	29 (39.20)
Type of stroke	
Ischemic	47 (63.51)
Hemorrhagic	27 (36.49)
Lesion location	
Brain stem	41 (55.40)
Non-brain stem	33 (44.60)
Dietary method	
Tube feeding	11 (14.86)
Oral feeding	63 (85.14)
MBI	43.34 ± 24.80
MMSE	23.50 ± 2.15
VSA	25.39 ± 15.66
FCR	1.03 ± 0.22
VDS	29.03 ± 15.54

Values are presented as the mean ± standard deviation or number (%). MBI, modified Barthel index; MMSE, Mini-Mental State Examination; VSA, vowel space area; FCR, formant centralization ratio; VDS, videofluoroscopic dysphagia scale; yr, years.

**Table 2:** Correlation analysis of the VDS with age, VSA, and FCR.

	VDS	*p*-Value
VSA	r = −0.760 **	<0.001
FCR	r = 0.417 *	0.001
Age	r = 0.238 *	0.041

VSA, vowel space area; FCR, formant centralization ratio; *r*, correlation coefficient; VDS, videofluoroscopic dysphagia scale. * *p* < 0.05, ** *p* < 0.001.

**Table 3:** Multiple linear regression analysis of factors correlated with the VDS.

	Standardized *β*	*p*-Value	Adjusted *R*^2^
VSA	−0.760 **	<0.001	0.571
FCR	0.083	0.373	
Age	0.025	0.776	

The variables were based on the order of listing in multiple linear regression analysis. VSA, vowel space area; FCR, formant centralization ratio. ** *p* < 0.001.

**Table 4:** Patient characteristics based on the VSA.

	Group A (*n* = 13)	Group B (*n* = 20)	Group C (*n* = 18)	Group D (*n* = 13)	Group E (*n* = 10)	Scheffe’s Test	F	*p*-Value
VSA	49.60 ± 5.80	30.33 ± 4.81	16.27 ± 3.57	5.97 ± 3.06				
VDS	10.73 ± 7.67	19.45 ± 7.25	31.94 ± 7.70	52.00 ± 8.77	36.90 ± 4.43	A<B<C,E<D	62.479	*p* < 0.001 **
FCR	0.91 ± 0.12	0.96 ± 0.13	1.09 ± 0.28	1.18 ± 0.21	1.57 ± 0.46	A,B,C,D<E	12.388	*p* < 0.001 **
Age (yr)	59.85 ± 11.17	64.95 ± 13.31	66.44 ± 14.42	73.77 ± 11.64	72.50 ± 10.56		2.583	*p* = 0.045 *

Values are presented as mean ± standard deviation. Groups A to D were grouped according to VSA, and Group E was a group with distortion of the vowel space. VSA, vowel space area; FCR, formant centralization ratio; VDS, videofluoroscopic dysphagia scale; yr, years. * *p* < 0.05, ** *p* < 0.001.

## Data Availability

The datasets generated and/or analyzed during the current study are available from the corresponding author on reasonable request.
